# Supplementary material for: Exploratory mass cytometry analysis reveals immunophenotypes of cancer treatment-related pneumonitis
Source: eLife. 2024 Apr 12;12:RP87288. doi: 10.7554/eLife.87288 (PMC11014725; doi:10.7554/eLife.87288)
Supplement: Supplementary file 2 [file elife-87288-supp1.zip › Supplementary file 1/Supplementary file 1.odt]

Supplemental Table 1. Mass cytometry antibody panels.

For CD45 barcoding

| Label | Target | product_id | clone | Amount (uL) |
| --- | --- | --- | --- | --- |
| 89Y | CD45 | 3089003B | HI30 | 1 |
| 106Cd | CD45 | 3106001C | HI30 | 1 |
| 110Cd | CD45 | 3110001C | HI30 | 1 |
| 112Cd | CD45 | 3112001C | HI30 | 1 |
| 116Cd | CD45 | 3116001C | HI30 | 1 |
| 196Pt | CD45 | 3196001C | HI30 | 1 |

For antibody cocktail #1 (T cell panel)

| Label | Target | Product ID | Clone | Amount (uL) |
| --- | --- | --- | --- | --- |
| 141Pr | CD3 | 3141019B | UCHT1 | 1 |
| 142Nd | CD11a | 3142006B | HI111 | 0.5 |
| 143Nd | CD5 | 3143007B | UCHT2 | 1 |
| 145Nd | CD4 | 3145001B | RPA-T4 | 1 |
| 146Nd | CD8a | 3146001B | RPA-T8 | 1 |
| 147Sm | CD7 | 3147006B | CD7-6B7 | 1 |
| 148Nd | CD274 (PD-L1) | 3148017B | 29E.2A3 | 1 |
| 149Sm | CD25 (IL-2R) | 3149010B | 2A3 | 1 |
| 150Nd | CD134 (OX40) | 3150023B | ACT35 | 1 |
| 151Eu | CD2 | 3151003B | TS1/8 | 1 |
| 152Sm | CD95/Fas | 3152017B | DX2 | 1 |
| 153Eu | TIM-3 | 3153008B | F38-2E2 | 1 |
| 154Sm | TIGIT | 3154016B | MBSA43 | 1 |
| 155Gd | CD279 (PD-1) | 3155009B | EH12.2H7 | 1 |
| 156Gd | CD183 (CXCR3) | 3156004B | G025H7 | 1 |
| 158Gd | ST2 (R&D AF523) | Labeling (201158A) |  | 1 |
| 159Tb | CD197 (CCR7) | 3159003A | G043H7 | 1 |
| 160Gd | CD28 | 3160003B | CD28.2 | 1 |
| 161Dy | CD152 (CTLA-4) | 3161004B | 14D3 | 1 |
| 162Dy | CD69 | 3162001B | FN50 | 1 |
| 163Dy | CD272 (BTLA) | 3163009B | MIH26 | 1 |
| 164Dy | CD45RO | 3164007B | UCHL1 | 1 |
| 165Ho | CD223/LAG-3 | 3165037B | 11C3C65 | 1 |
| 166Er | CD44 | 3166001B | BJ18 | 1 |
| 167Er | CD27 | 3167002B | O323 | 1 |
| 168Er | CD278/ICOS | 3168024B | C398.4A | 1 |
| 169Tm | CD19 | 3169011B | HIB19 | 1 |
| 170Er | CD45RA | 3170010B | HI100 | 1 |
| 171Yb | CD226 | 3171013C | DX11 | 1 |
| 172Yb | CD273 (PD-L2) | 3172014B | 24F.10C12 | 1 |
| 173Yb | HLA-DR | 3173005B | L243 | 0.5 |
| 174Yb | CD49d | 3174018B | 9F10 | 1 |
| 175Lu | CD14 | 3175015B | M5E2 | 2 |
| 176Yb | CD57 | 3176019B | HCD57 | 1 |
| 209Bi | CD16 | 3209002B | 3G8 | 1 |

For antibody cocktail #2 (B cell/ myeloid cell panel)

| Label | Target | Product ID | Clone | Amount (uL) |
| --- | --- | --- | --- | --- |
| 141Pr | CD3 | 3141019B | UCHT1 | 1 |
| 142Nd | CD19 | 3142001B | HIB19 | 0.5 |
| 143Nd | CD11b (Biolegend #301302) | Labeling(201143A) | ICRF44 | 0.5 |
| 144Nd | CD38 | 3144014B | HIT2 | 1 |
| 145Nd | CD163 | 3145010B | GHI/61 | 1 |
| 146Nd | CD64 | 3146006B | 10.1 | 1 |
| 147Sm | CD11c | 3147008B | Bu15 | 0.5 |
| 148Nd | IgA | 3148007B | Polyclonal | 1 |
| 149Sm | IgG (Biolegend #410701) | Labeling(201149A) |  | 1 |
| 150Nd | CD138 | 3150012B | DL-101 | 0.5 |
| 151Eu | CD209 (Biolegend #343002) | Labeling(201209A) |  | 1 |
| 152Sm | CD21 | 3152010B | BL13 | 1 |
| 153Eu | CD192 (CCR2) | 3153023B | K036C2 | 1 |
| 154Sm | CD84 | 3154013B | CD84.1.21 | 1 |
| 156Gd | CD86 | 3156008B | IT2.2 | 1 |
| 158Gd | ST2 (R&D AF523) | Labeling (201158A) |  | 1 |
| 159Tb | CD36 (Biolegend #336215) | Labeling(201159A) | 5-271 | 1 |
| 160Gd | CD28 | 3160003B | CD28.2 | 0.5 |
| 164Dy | CD185/CXCR5 | 3164029B | RF8B2 | 1 |
| 165Ho | CD223/LAG-3 | 3165037B | 11C3C65 | 1 |
| 166Er | CD24 | 3166007B | ML5 | 1 |
| 167Er | CD27 | 3167002B | O323 | 1 |
| 168Er | CD206 (MMR) | 3168008B | 44607 | 1 |
| 169Tm | CD32 | 3169020B | FUN-2 | 1 |
| 170Er | TIM-1 (Biolegend #353902) | Labeling(201170A) |  | 1 |
| 171Yb | CD195 (CCR5) | 3171017A | NP-6G4 | 1 |
| 172Yb | IgM | 3172004B | MHM-88 | 1 |
| 173Yb | HLA-DR | 3173005B | L243 | 0.5 |
| 174Yb | IgD (Biolegend #348235) | Labeling(201174A) | IA6-2 | 1 |
| 175Lu | CD14 | 3175015B | M5E2 | 2 |
| 176Yb | antiAPC-176Yb | 3176007B | APC003 | 1 |
| 209Bi | CD16 | 3209002B | 3G8 | 1 |
